# Safe Drinking Water, Sanitation and Mother's Hygiene Practice as Stunting Risk Factors: A Case Control Study in a Rural Area of Ciawi Sub-district, Tasikmalaya District, West Java, Indonesia

**DOI:** 10.4314/ejhs.v33i6.3

**Published:** 2023-11

**Authors:** Siti Novianti, Emy Huriyati, Retna Siwi Padmawati

**Affiliations:** 1 Doctoral Program in Medicine and Health Science, Faculty of Medicine, Public Health and Nursing, Universitas Gadjah Mada, Jl. Farmako, Senolowo, Sekip Utara, 55281 Sleman Yogyakarta, Indonesia; 2 Department of Nutrition and Health, Faculty of Medicine, Public Health and Nursing, Universitas Gadjah Mada, Yogjakarta Indonesia; 3 Department of Health Behavior, Environment, and Social Medicine, Faculty of Medicine, Public Health and Nursing, Universitas Gadjah Mada, Yogyakarta, Indonesia

**Keywords:** Drinking water, sanitation, hygiene practices, stunting, children

## Abstract

**Background:**

Stunting is associated with disorders of the small intestines caused by environmental factors and poor hygiene practices. Prevention of stunting should be conducted in the first 1,000 days of life; namely, from conception until the child is two years old. This research aimed to analyze the relationship between environmental risk factors and maternal personal hygiene with the incidence of stunting in children aged 6-23 months.

**Methods:**

This study was conducted using a case-control design, with a total sample of 212 (106 cases and 106 controls) enrolled purposively. Data were collected through interviews using a questionnaire. The analysis used chisquare tests and multiple logistic regression.

**Results:**

The results of multivariate analysis showed the independent variables that have a significant association on the incidence of stunting are access to safe drinking water and maternal hygiene practices. The external variables that have a significant relationship is birth length and feeding practice.

**Conclusion:**

Having no access to safe drinking water, not proper sanitation, and poor maternal hygiene practices have a higher risk of stunting in children aged 6-23 months. The implications of this research include the need for increased access to a safe environment and improvement of mother's behavior as essential efforts to prevent stunting.

## Introduction

Stunting is a manifestation of malnutrition and an important health problem. Although the global prevalence of stunting tends to decline between 2000-2022, Joint Malnutrition Estimates (JME) release in 2023 reveals not enough progress towards achieving World Health 2025 Global Assembly (WHA) nutrition targets (1,2). In most children, the stunting incidence starts before birth as a result of the low nutritional status of pregnant women and gradually gets worse during the first two years of life (3). The result of a systematic review reported that the risk factors of undernutrition in children were child age, child sex, complementary food, diarrheal disease, dieteary diversity, maternal education, maternal height, living in rural area and socioeconomic status (4).

Stunting is a cyclical process because women who are short in their childhood tend to give birth to short children, creating an intergenerational cycle of poverty and reducing human resources that are difficult to solve (5). The key of stunting prevention starts from the period of conception until the age of two, known as the First 1,000 Days of Life. After the age of two years, the growth rate tends to decrease and the child will be considered stunted (1,6).

Assuming that children's low growth is caused by children who are not eating enough and lack of good nutrition, many studies focused on identifying improved nutrition as a solution (7). The results of the research by Bhutta (8) estimated that a hygiene and sanitation intervention with 99% coverage can reduce the incidence of diarrhea by 30%, but the effect on reducing stunting prevalence is only 2-4%. The results of the research also explained that the key of causing malnutrition in children is a subclinical disorder of the small intestines known as Environmental Enteric Dysfunction (EED). EED is a chronic inflammation that causes histologic changes in the small intestines, where this disorder occurs in individuals who live in tropical areas (9). This condition occurs in environments that have poor hygiene and sanitation, and often happens in developing countries due to constant fecal-oral contamination (10,11).

Efforts of improving sanitation and hygiene, simply known as WASH (Water, Sanitation and Hygiene) have a positive effect on reduction and prevention of EED and will also have an impact on decreasing linear growth disorders (5,11). Based on the WHO report, studies regarding the impact of WASH on diseases other than diarrhea are still limited (6).

According to several recent findings, improving access to sanitation is linked to a decline in the prevalence of stunting (3,14,15). According to Basic Health Research Data (Riskesdas) and the 2022 Indonesian Nutrition Status Survey (SSGI), West Java province is the highest prevalence of stunting, 33.3%, included Tasikmalaya district with prevalence rate 29.2% higher than the national average (22.3%). Since 2020, Ciawi subdistrict has been designated as a priority region for prevention (16–18).

Numerous studies have linked environmental factors to the prevalence of a number of environmental-based disorders, including helminthiasis and diarrhea. However, there is still little information available on how environmental factors affect childhood development abnormalities, such as stunting. This study aims to examine the association between maternal hygiene practices, access to sanitation, and the incidence of stunting in infants aged 6 to 23 months.

## Methods

**Study design and setting**: This research used a case-control design, which aims to analyze the risk of access to safe drinking water, access to proper sanitation and maternal hygiene practices related with the stunting in children aged 6-23 months. The research was conducted in Ciawi sub-district, the working area of Ciawi Public Health Center, Tasikmalaya district. Stunting has been prevalent in Ciawi sub-district over the past three years, with a prevalence of above 20%. Children are defined as stunted if their height-forage is more than two standard deviations below the WHO Child Growth Standards median (19).

The research subjects were children aged 6-23 years who were registered in the weighing month data for toddlers with a total sample of 212 (106 cases and 106 controls). Stunted children were identified based on the results of routine weighing in Posyandu by health cadres and validated by midwives. This data is also a source of reporting from the puskesmas to the health office of Tasikmalaya district (Dinas Kesehatan) as a basis for determining the locus of stunting. Based on the results of interviews with midwives as anthropometric data validators, height measurements use a lengthboard. Cases were all stunting children and controls were children with good nutritional status, who were the closest neighbors of cases and were taken purposively. Respondents were mothers who were caregivers for children and live together. This study was approved by the Medical and Health Research Ethics Committee (MHREC), Faculty of Medicine, Public Health and Nursing, Universitas Gadjah Mada – Dr. Sardjito General Hospital with the numbers - Ref : KE/FK/1343/EC/2020 and Ref : KE/FK/1343/EC/2021. Informed consent was obtained from all participants of this study.

**Data collection and analysis**: Data were collected through observations and interviews by using a structured questionnaire. The data collectors were researchers (SN) and assisted by enumerators (students of public health and had been trained) and under the supervision of RSP and EH. The variables measured were sociodemographics and maternal health history during pregnancy, history of the child at birth, exclusive breastfeeding practices, feeding practices, and history of infection (diarrhea). Maternal health including consumption of iron tablets during pregnancy, and history of disease during pregnancy (tuberculosis infection and anemia). Programs in Indonesia require pregnant women to consume 90 tablets of iron during pregnancy. Variable categorized “no” if the mother did not consume 90 iron tablets during pregnancy, and “yes” if the mother consumed 90 iron tablets during her pregnancy. Variable history of the child at birth are low birth weight (defined as the birth weight is less than 2500 grams) and normal weight (if the birth weight is ≥ 2500 grams) and birth length (categorized less than 48 centimeters and ≥ 48 centimeters). Exclusively breastfeeding is defined as the practice of only giving an infant breast-milk for the first 6 months of life (no other food or water). Feeding practices is define as mother's practice in child feeding which includes early initiation of breastfeeding, exclusive breastfeeding, various complementary feeding, type and frequency of feeding, and responsive feeding (19,20).

Access to drinking water and sanitation used the algorithm list of BAPPENAS which is a proxy/adaptation of the WHO/JMP questionnaire. The criteria for access to safe drinking water are that the main drinking water source comes from a protected drinking water source, is located on premises, takes <10 minutes to collect water, and meets the physical requirements (colorless, odorless, and tasteless). The criteria for access to sanitation are having a latrine used by the members of household, having a gooseneck latrine, and having a septic tank (22–24). The questionnaire on maternal hygiene practices used the WHO/UNICEF JMP criteria, consists of the practice of washing hands at five important times and the proper disposal of baby feces (22–24). Data related to maternal health status and birth history were obtained from the Maternal and Child Health Book (KIA Book). Data were analyzed descriptive, bivariate (Chi-square test) and multivariate with logistic regression. The candidate for multivariate analysis were variables had a *p* value ≤ 0.25. The data was analyzed by using a computer software (SPSS UGM license) with a significance level of 0.05 and 95% confidence level (CI).

## Results

The characteristic of respondence shown in Table 1. Most of the respondents had a low level of education (elementary school graduates). A total of 10.4% of stunted group had mothers with a history of anemia, and 4.7% had a history of pulmonary TB infection.

**Table 1:** Sociodemographic of Respondents in Ciawi Sub-district, Tasikmalaya District, West Java, Indonesia

Variable	Stunting		Not Stunting		*p value*

	f	%	f	%	
Mother's education level					
Elementary school	40	37.7	41	38.7	0.237
Junior high school	45	42.5	36	34.0	
Senior high school	16	15.1	26	24.5	
Diploma/bachelor	5	4.7	3	2.8	
Mom's job					
Housewife	105	98.2	104	98.6	1.000
Working mom's	2	1.8	2	1.4	
Father's education level					
Elementary school	52	49.0	45	42.4	0.459
Junior high school	27	25.5	35	33.0	
Senior high school	27	25.5	26	24.6	
Parity					
1	29	27.4	31	29.2	0.583
2	34	32.1	41	38.7	
3	31	29.2	23	21.7	
≥4	12	11.3	11	10.4	
Ante Natal Care during Pregnancy					
< 4 times	16	15.1	19	17.9	0.711
≥4 times	90	84.9	87	82.1	
Places of delivery					
Non health facility	16	15.1	19	17.9	0.579
Health facility	90	84.9	87	82.1	
Tuberculosis infection during pregnancy					
Yes	5	4.7	9	8.5	
No	101	95.3	97	81.5	0.407
Anemia during pregnancy					
Anemia	11	10.4	12	11.3	0.239
Non anemia	35	33.0	24	22.6	
Not measured	60	56.6	70	66.0	

The results of the analysis the variables with the Chi-square tests seen in Table 2 shows that there were three variables: access to safe drinking water, access to proper sanitation and maternal hygiene practices which had a significant relationship with the incidence of stunting in children aged 6-23 months. The results of the bivariate analysis on the external variables identified a significant relationship with birth length and feeding practices. Meanwhile, the other external variables were not statistically significant (*p*>0.05). Based on the last model (Model 4 in Table 3), it is known that the variable access to proper sanitation facilities becomes insignificant after controlling the external variables and birth length as the dominant factors with aOR 4.0. The fourth model was chosen because the parameter estimates change is less than 0.1. Therefore, an R^2^ value of 22% means that access to safe drinking water, mother's hygiene, birth length and feeding practice can predict the incidence of stunting in children aged 6-23 months as much as 22%.

**Table 2:** Factors Independently Associated with Stunting Among 6 to 23 month-age-children in Ciawi Sub-district, Tasikmalaya Distric, West Java, Indonesia, 2023 (based on bivariate analysis)

Variable	Stunting		Not Stunting		*p* value	95% CI	cOR

	f	%	f	%			
Access to safe drinking water							
No access (ref)	85	80.2	61	57.5	0.001*	1.617-5.515	2.9
Yes	21	19.8	45	42.5			
Access to proper sanitation							
No access (ref)	63	59.4	46	43.4	0.028*	1.107-3.298	5.4
Yes	43	40.6	60	56.6			
Mother's hygiene practice							
Poor (ref)	52	49.1	36	34.0	0.037*	1.076-3.257	1.87
Good	54	50.9	70	66.0			
Family income							
<regional min. wage (IDR 2.223.000)							
(ref)	60	56.6	59	55.7	1.000	0.604-1.788	1.039
≥ regional min. wage	46	43.4	47	44.3			
Parity							
Nullipara and grande multipara (ref)	41	38.7	41	38.7	1.000	0.575-1.758	1.00
Multipara	65	61.3	65	61.3			
Mother's age at pregnancy							
< 20 years and/or ≥ 35 years (ref)	31	29.2	33	31.1	0.881	0.509-1.644	0.914
20 - 35 years	75	70.8	73	68.9			
Consumption of iron tablet during pregnancy (90 pils)							
No (ref)	31	29.2	43	40.6	0.082	0.340-1.069	0.602
Yes	75	80.8	63	59.4			
LBW (birth weight < 2500 gram)							
Yes (ref)	7	6.6	2	1.9	0.170	0.746-18.127	3.67
No	99	93.4	104	98.1			
Birth length							
< 48 cm (ref)	26	24.5	2	1.9	0.03*	1.552-7.904	3.50
≥ 48 cm	80	75.2	104	98.1			
Early initiation of breastfeeding							
No (ref)	56	52.8	53	50.0	0.783	0.653-1.920	1.120
Yes	50	47.2	53	50.0			
Exclusive breastfeeding							
No (ref)	36	34.0	27	25.5	0.229	0.367-1.203	0.665
Yes	70	66.0	79	74.5			
Complementary feeding							
Early (ref)	90	84.9	93	87.7	0.689	0.358-1.728	0.786
6 months	16	13.1	13	12.3			
Diarrheal infection in last two months							
Yes (ref)	31	29.2	32	30.2	1.000	0.530-1.723	0.956
No	75	70.8	74	69.8			
Feeding Practices							
Poor	37	34.9	52	49.1	0.037*	0.321-0.967	0.557
Good	69	65.1	54	50.9			

* Significant (*p* value < 0.05); CI, Confidence Interval; cOR, crude Odds Ratio.

**Table 3:** Multivariate analysis risk factor related stunting children aged 6-23 months in Ciawi Sub-district, Tasikmalaya District, West Java, Indonesia, 2023

Variable	SE	*P value*	aOR	95% CI	R^2^
**Model 1**					
Access to safe drinking water	0.342	0.001	3.2	1.626-6.213	0.232
Access to proper sanitation	0.310	0.188	1.5	0.819-2.758	
Mother's hygiene practice	0.323	0.018	2.1	1.137-4.033	
Feeding practice	0.335	0.032	0.4	0.253-0.941	
Consumption of iron tablet	0.326	0.059	0.5	0.285-1.023	
Low birth weight	0.886	0.560	1.7	0.295-9.523	
Birth length	0.468	0.006	3.5	1.432-8.975	
Exclusively breastfeeding	0.419	0.841	1.1	0.552-2.075	
**Model 2**					
Access to safe drinking water	0.341	0.001	3,2	1.635-6.229	0.231
Access to proper sanitation	0.309	0.192	1,5	0.817-2.741	
Mother's hygine practice	0.322	0.018	2,1	1.143-4.044	
Feeding practice	0.321	0.022	0,5	0.255-0.897	
Consumption of iron tablet	0.324	0.060	0,5	0.288-1.027	
Low birth weight	0.885	0.563	1,7	0.295-9.443	
Birth length	0.467	0.007	3,6	1.424-8.871	
**Model 3**					
Access to safe drinking water	0.341	0.001	3,2	1.645-6.255	0.230
Access to proper sanitation	0.306	0.164	1,5	0.841-2.790	
Mother's hygiene practice	0.321	0.019	2,1	1.131-3.980	
Feeding practice	0.320	0.020	0,5	0.253-0.997	
Consumption of iron tablet	0.324	0.060	3,8	0.288-1.025	
Birth length	0.453	0.003	0,4	1.571-9.275	
**Model 4**					
Access to safe drinking water	0.338	0.000	3.3	1.724-6.479	0.219
Mother's hygiene practice	0.317	0.011	2.2	1.204-4.180	
Feeding practice	0.317	0.021	0.5	0.258-0.895	
Consumption of iron tablet	0.322	0.060	0.5	0.291-1.027	
Birth length	0.453	0.002	4.0	1.654-9.745	

aOR, adjusted Odds Ratio

## Discussion

The results of this research showed that the absence of access to safe drinking water increased the risk of stunting by 2.9 times in children aged 6-23 months. A total of 67.7% of the stunted group and 69.8% of the non-stunted group had the category of protected drinking water sources. It means that most of the research subjects already had access to basic drinking water (basic access). However, the distance proportion of the springs' sources to the waste disposal site was less than 10 meters, which is greater than the ideal distance, which includes more than 10% in the stunted and non-stunted groups.

This finding is in line with the research in rural areas of Ethiopia (25) and Basic Health Research (Riskesdas) in Indonesia (16), found that protected water sources, protected drinking water sources and distance to sources of pollution were significantly associated with the incidence of stunting in children under the age of five. Although the physical quality of water in this research was not significant, it was found that there was 1.2 times higher tendency to experience stunting in toddlers with poor physical water quality.

The concept of drinking water is in accordance with Permenkes number 492/Menkes/PER/IV/2010. A household which uses a proper drinking water source, the water source location is inside or on the premises, available whenever needed and the quality of drinking water based on physical parameters categorized as safe drinking water (22,26).

The 2015-2018 BPS Susenas which was conducted by BAPPENAS showed the achievement of access to safe water nationally in 2018 reached 61.29%, which increased compared to 2017 which was 59.07% (22). Data from the Ministry of Health in 2020 concerning the results of a study on the quality of household drinking water showed that access to safe drinking water in Indonesia was 93%, consisting of 97.6% in urban areas and 87.1% in rural areas. However, only 11.9% already had access to safe drinking water; 15.3% in urban areas and 8.3% in rural areas. As many as 7 out of 10 households in Indonesia consume drinking water from facilities which are contaminated with *E. coli* (27).

The unavailability of proper sanitation facilities increases the risk of stunting by 5.4 times. This finding is in line with a research (16) which found that non-goose neck latrines had a 1.3 times risk of stunting. This is also in accordance with the research (28) which revealed that children < 2 who do not have access to good sanitation experience a height deficit of 0.9 cm. The height of children < 2 who had poor water sources was 1.0 cm shorter than children < 2 who had good water sources. Another research was conducted in India (29) on the relationship between open defecation and stunting in children. In addition, the results of research in developing countries and Southeast Asia showed that poor sanitation related with stunting in children < 5 (30).

Lack of access to sanitation facilities, leads to various health challenges such as helminth infections and enteropathy. Environmental enteropathy occurs due to repeated and long-term inflammation of the small intestines which results in reduced ability to absorb nutrients and causes health problems such as anemia, diarrhea and stunting (31). A household is considered to have access to proper sanitation if it meets several components, such as using a goose-neck toilet, having a septic tank or a Waste Water Treatment System (SPAL) and/or ground pit (for rural areas), and being used by the household itself or together with certain other households.

There is a slight difference in the criteria for sanitation facilities in rural areas; with a goose neck model where the final disposal of feces may use the ground pit that is categorized as access to proper sanitation. This is in accordance with the approach to sanitation development policies that still accommodate simple/basic sanitation facilities which are built by the community independently in areas with low population density. If the household does not have sanitation facilities or have sanitation facilities but do not use it, these are included in the open defecation behavior (22).

The results of a research conducted by Danaei (32) found that 72% cases of stunting in the worldwide were caused by poor sanitation. The burden that arises from inadequate sanitation for the occurrence of stunting is greater than diarrhea in toddlerhood. Research in Bengkulu showed that poor environmental sanitation had 3.8 times greater risk of the incidence of stunting. (17) Hygiene and sanitation interventions with 99% coverage can reduce the incidence of diarrhea by 30%, but the effect on reducing stunting prevalence is 4-37% in children < 5 in urban areas and 20-46% in rural areas. Meanwhile, the provision of nutritional intervention only had an effect of 15.5-21.7% (8).

According to UNICEF, inadequate latrine facilities and unsafe drinking water quality have the risk to increase the percentage of children < 5 with poor nutrition and contribute to the already high number of stunted children in Indonesia. The implications for the policy are already clear, i.e., the efforts of overcoming the problem of malnutrition need to be accompanied by improvements in drinking water, sanitation and hygiene conditions (33). Access to safe drinking water, adequate sanitation facilities, clean and healthy living behavior are the parameters that determine the health status of the Indonesian population. Research concerning the determinants of stunting in Indonesia also showed a connection between access to water and sanitation with the occurrence of stunting (30,34,35).

The efforts of improving sanitation and hygiene, through the provision of latrines and increasing hand washing behavior after contact with feces, simply known as WASH (Water, Sanitation and Hygiene), have an effect on reducing and preventing EED and will also have an impact on decreasing linear growth disorders (3,7,12,13). Lack of access to clean and proper drinking water and poor environmental sanitation are important causes of malnutrition. This condition directly affects health, food production and general hygiene. Poor access to clean water and inadequate drinking water also indirectly affects nutrition through an increase in the workload of women that will affect the time available to take care of their children (36,37).

Children whose mothers have poor hygiene practices are 1.9 times more likely to have stunting. According to Humphrey (2009), the main factor contributing to childhood malnutrition is a subclinical condition of the small intestine known as tropical enteropathy, which is brought on by children ingesting excessive amounts of excrement carrying bacteria when they live in unsanitary and unhygienic settings. Toddlers can be safeguarded against fecal contamination by safely disposing of their waste (defecating in latrines) and washing their hands with soap after coming into touch with feces as the primary defense against fecal-oral transmission (7,13).

According to studies conducted in rural Ethiopia (25) and also in line with another research (38), mothers who do not wash their hands before eating, do not wash their hands after going to the bathroom, and do not wash their hands with soap both after going to the bathroom and before eating are predictors of the incidence of stunting in toddlers. A crucial component of childcare is maintaining good maternal hygiene because the mother is the main caregiver for the kids.

However, the efforts to increase maternal hygiene practices through campaigns on the importance of washing hands with soap will not run effectively if there is no access to proper clean water. Further, the unavailability of handwashing facilities and soap in the toilets is one of the causes of the difficulty in practicing hand washing at important times as recommended. As in this study, due to limited socio-economic conditions, where the most of the case and control groups have an income below the regional minimum wage.

In conclusion, the main risk factors for stunting in children aged 6–23 months were lack of access to safe drinking water, mother's hygiene practices, feeding practices, and birth length. This study illustrates that environmental factors, although they are indirect causes, have a significant influence. The government does have an important role to play in ensuring that people have access to drinking water and proper sanitation facilities. However, it is also important to encourage people to raise awareness and work independently to increase access to clean water and sanitation as well as improved hygiene practices. Besides, maternal quality to obtain better pregnancy outcomes with quality antenatal care services and feeding practices in the first two years of life, also known as the first 1000 days of life, is also an important part of preventing stunting and making future generations better.

There are many studies focusing on efforts to increase adequate food intake. The strength of this study is that it measured environmental factors as part of the multifactorial causes of stunting. Besides, in order to answer the research question and identify potential risk factors for stunting in children aged 6 to 23 months, a case-control study design was used.

Yet, there were some limitations in our study. It is challenging to extrapolate findings from small samples to the entire population. We were unable to collect information regarding food intake recall and examine the biological quality of drinking water. If done, it might have provided us with more information.
